# Clinical remission after biologic therapy discontinuation in pediatric patients with severe asthma: a case series from a tertiary center

**DOI:** 10.36416/1806-3756/e20230405

**Published:** 2024-05-08

**Authors:** Giovana De Marchi Castelli, Frederico Friederich, Anasthácia Ferreira Wiemann, Giovana dos Santos, Paulo Márcio Pitrez

**Affiliations:** 1. Universidade Federal de Ciências da Saúde de Porto Alegre - UFCSPA - Porto Alegre (RS) Brasil.; 2. Pontifícia Universidade Católica do Rio Grande do Sul - PUCRS - Porto Alegre (RS) Brasil.

## TO THE EDITOR:

Given the complex interactions between genetic and environmental factors, asthma is a heterogeneous chronic lung disease, characterized by different patterns of inflammation and bronchial remodelling.^1^ The treatment aims at clinical disease control with optimized or normal lung function, applying different steps for treatment, from inhaled corticosteroids (ICS) as monotherapy to add-on therapies, including biologics. Severe asthma is a major clinical challenge, but the access to biologics resulted in better control of disease.^1^ In Brazil, omalizumab was the first biologic therapy approved for severe asthma in the pediatric population, demonstrating significant reductions in the number of exacerbations, in the use of rescue medications, and in emergency room visits during the first year of treatment, maintaining clinical improvement.^2^ ^,^ ^3^ These clinical effects arise the possibility of clinical remission on and off treatment, becoming an important scientific topic worldwide.^4^

Aiming to describe the characteristics and the course of clinical remission with omalizumab therapy discontinuation, we contacted patients from a previous cohort of Brazilian pediatric patients with severe asthma, aged 14-21 years, followed in a tertiary center. Between 2012 and 2018, the patients underwent a clinical protocol approach to diagnose severe asthma based on GINA criteria^5^ and used omalizumab as an add-on therapy. Of the 21 eligible patients with severe asthma, 8 agreed to participate in this follow-up case series (mean age = 17.2 ± 1.9 years; 4 were female). The patients were assessed through an in-person visit or a web conference. Patient-reported outcomes included clinical history, dose of medications, assessment of disease control (GINA questionnaire), and history of hospitalizations. For those patients who visited the tertiary center, pulmonary function was assessed by measuring FEV_1_ and FEV_1_/FVC ratio. Data were also collected from standardized electronic medical records. The study was approved by the research ethics committee of the institution (CAAE n. 50818220.0.0000.5345).

Lack of disease control was defined as the presence of poor symptom control (GINA criteria), > 2 severe or serious exacerbations in the previous year (with the use of oral corticosteroids [OCS] or hospitalization, respectively), or persistent airflow limitation.^1^ Clinical remission on- and off-treatment with omalizumab was defined by the sustained absence of significant symptoms (> 12 months of total control of disease and no exacerbations).^4^

**Figure 1:**
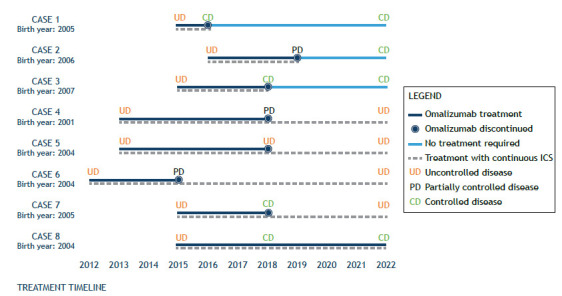
Evolution of clinical and disease control with omalizumab treatment. Each line on the Y-axis represents one individual of this case series. Data from medical records and interviews comprise a period of up to 10 years of registry. Outcomes are depicted for each individual at pre-treatment, post-treatment, and follow-up. ICS: inhaled corticosteroids.

All of the 8 patients were previously classified as having atopic severe asthma (positive skin-prick test), uncontrolled asthma symptoms, normal or mild airway obstruction, and a history of hospitalization in the year before the initiation of omalizumab therapy. Six patients showed allergic rhinitis, and 4 patients had as a comorbidity being overweight/obese. The mean duration of omalizumab treatment was 46 ± 25 months. The mean age at initiation of omalizumab therapy was 9.0 ± 1.6 years, and the mean age at discontinuation was 13.0 ± 2.9 years. Only 1 patient did not achieve clinical remission to discontinue the omalizumab treatment. The 7 patients who discontinued omalizumab had from 3 to 7 years off-treatment with omalizumab until the follow-up visit. Figure 1 shows disease control and the course of treatment for each patient. After omalizumab prescription, hospital admissions were significantly reduced, from pre-treatment, post-treatment to follow-up, with no report of hospital admission in the last 12 months before data collection.

On clinical follow-up, 4 of the 7 patients who discontinued omalizumab reported uncontrolled disease. All patients are currently on continuous ICS. The mean daily dose of ICS was significantly reduced from pre-treatment to follow-up (at pre-treatment: 537.5 μg/day; at post-treatment: 425.0 μg/day; and at follow-up: 212.5 μg/day; p = 0.0179-comparisons performed with Friedman test). Also, symptom control was significantly improved after omalizumab treatment; 1 patient reported partially controlled symptoms, and 1 reported uncontrolled symptoms (a 40% reduction; p = 0.0373). The 4 patients originally on continuous OCS discontinued this treatment by six weeks after initiating omalizumab. No serious adverse events from omalizumab were observed. Lung function, considered a less important tool for assessing the severity of asthma in children,^6^ was not significantly changed by omalizumab treatment for all variables analyzed during pre-treatment, post-treatment, and follow-up (comparisons using mixed effect analysis).

Three patients (42.8%) are in clinical remission 4-6 years after discontinuing omalizumab. In their medical records, all of these patients had a history of multiple hospitalizations before omalizumab treatment. This is probably the most important clinical goal for any severe presentation of a chronic disease.

One previous real-life study including our case series showed that omalizumab significantly reduced hospitalizations, OCS use, and improved disease control.^7^ In this report, we demonstrated that, after omalizumab discontinuation, 3 of the 8 patients achieved long-term total control of disease (off-treatment clinical remission), being treated only with inhaled controller medications. However, despite preventing further hospitalizations in all cases, 4 patients did not show clinical remission off-treatment, but none of them returned to show a severe phenotype.

The first real-life study of omalizumab discontinuation conducted with 35 children showed that patients with well-controlled disease generally maintained disease control when the drug was discontinued. However, 22% had to resume omalizumab therapy due to worsening of symptoms.^8^ Two other large cohort studies with pediatric patients with severe asthma showed that long-term off-treatment clinical remission after the use of omalizumab was observed in 27% (n = 100) and in 33% (n = 1,082) of the patients, respectively.^8^ ^,^ ^9^ Our report also shows, in a Brazilian population, that clinical remission after discontinuation of a biologic in pediatric patients with severe asthma may not be an uncommon outcome. Despite the limitations of this report, because data were retrospectively collected from medical records, and a self-report questionnaire regarding the previous 12 months was applied, which is dependent on patient and parent recall, we were able to demonstrate that long-term clinical off-treatment remission of asthma after omalizumab use may be achieved in the management of Brazilian pediatric patients with severe asthma.

Off-treatment clinical remission is one of the most important clinical targets in asthma, and our case series of pediatric patients suggests that this outcome may be achieved in almost half of the patients with severe asthma treated with biologics. Large national multicenter prospective cohort studies in pediatric patients with severe asthma are urgently needed to better define off-treatment asthma remission, the prevalence of this outcome, and predictors of success.
